# Nominal carbonic anhydrase activity minimizes airway‐surface liquid pH changes during breathing

**DOI:** 10.14814/phy2.13569

**Published:** 2018-01-25

**Authors:** Ian M. Thornell, Xiaopeng Li, Xiao Xiao Tang, Christian M. Brommel, Philip H. Karp, Michael J. Welsh, Joseph Zabner

**Affiliations:** ^1^ Department of Internal Medicine University of Iowa Iowa City IA; ^2^ Howard Hughes Medical Institute University of Iowa Iowa City IA; ^3^ Department of Molecular Physiology and Biophysics University of Iowa Iowa City IA

**Keywords:** Acid‐base, airway epithelium, airway‐surface liquid, carbonic anhydrase

## Abstract

The airway‐surface liquid pH (pH_ASL_) is slightly acidic relative to the plasma and becomes more acidic in airway diseases, leading to impaired host defense. CO_2_ in the large airways decreases during inspiration (0.04% CO_2_) and increases during expiration (5% CO_2_). Thus, we hypothesized that pH_ASL_ would fluctuate during the respiratory cycle. We measured pH_ASL_ on cultures of airway epithelia while changing apical CO_2_ concentrations. Changing apical CO_2_ produced only very slow pH_ASL_ changes, occurring in minutes, inconsistent with respiratory phases that occur in a few seconds. We hypothesized that pH changes were slow because airway‐surface liquid has little carbonic anhydrase activity. To test this hypothesis, we applied the carbonic anhydrase inhibitor acetazolamide and found minimal effects on CO_2_‐induced pH_ASL_ changes. In contrast, adding carbonic anhydrase significantly increased the rate of change in pH_ASL_. Using pH‐dependent rates obtained from these experiments, we modeled the pH_ASL_ during respiration to further understand how pH changes with physiologic and pathophysiologic respiratory cycles. Modeled pH_ASL_ oscillations were small and affected by the respiration rate, but not the inspiratory:expiratory ratio. Modeled equilibrium pH_ASL_ was affected by the inspiratory:expiratory ratio, but not the respiration rate. The airway epithelium is the only tissue that is exposed to large and rapid CO_2_ fluctuations. We speculate that the airways may have evolved minimal carbonic anhydrase activity to mitigate large changes in the pH_ASL_ during breathing that could potentially affect pH‐sensitive components of ASL.

## Introduction

Respiratory epithelia are covered by a thin ~10 *μ*m layer of liquid termed the airway‐surface liquid (ASL) (Widdicombe and Wine 2015). The ASL pH (pH_ASL_) is typically a few tenths of a pH unit more acidic than the plasma pH (Jayaraman et al. 2001; Coakley et al. 2003; McShane et al. 2003; Song et al. 2006; Pezzulo et al. 2012; Garland et al. 2013; Abou Alaiwa et al. 2014a). pH_ASL_ maintenance is vital to respiratory host defense by influencing ASL antimicrobial activity (Pezzulo et al. 2012; Abou Alaiwa et al. 2014b; Shah et al. 2016), mucociliary transport (Clary‐Meinesz et al. 1998; Tang et al. 2016), and phagocyte function (Trevani et al. 1999). Abnormalities in these pH_ASL_‐dependent functions likely contribute to several pathological conditions such as cystic fibrosis (Smith and Welsh 1992, 1993; Hug et al. 2003; Quinton 2008; Chen et al. 2010a; Itani et al. 2011), asthma (Hunt et al. 2000; Kostikas et al. 2002), chronic obstructive pulmonary disease (Kostikas et al. 2002), and acute respiratory distress syndrome (Gessner et al. 2003; Walsh et al. 2006), where pH_ASL_ is acidified relative to nondisease ASL.

The pH_ASL_ is influenced by a number of H^+^‐ and HCO_3_^–^‐dependent mechanisms (Fischer and Widdicombe 2006). HCO_3_^–^ is a powerful buffer because it is in an open‐buffering system with CO_2_, following the elementary equilibrium reaction:

$${\text{CO}}_{2}+H_{2}O↔H^{+}+{\text{HCO}}_{3}^{-}$$

Within the cytoplasm and some extracellular fluid compartments, the CO_2_/HCO_3_^–^ buffering system is catalyzed to a nearly diffusion‐limited rate by carbonic anhydrase, 1.6 × 10^6^ sec^−1^ for carbonic anhydrase isozyme II (Supuran 2008). For most cell types and compartments, the CO_2_ concentration is a nearly constant 5%. However, the CO_2_ concentration in large proximal airways changes during the respiratory cycle (Cochrane et al. 1982). This change is observed in a clinical setting by variations in end‐tidal CO_2_. During inspiration (1–2 sec), the CO_2_ concentration in the large airway lumen approaches that of air (0.04% CO_2_), while during expiration (2–4 sec) the CO_2_ concentration approaches 5%.

If carbonic anhydrase is present in the ASL, then fast CO_2_ equilibration will change pH_ASL_ in the large airways during breathing. Here, we tested this prediction using differentiated cultures of porcine airway epithelia. We were surprised to discover that changing the CO_2_ concentration between 0.04% and 5% produced little change in pH_ASL_ over a time course associated with respiration (i.e., a few seconds). We found that nominal carbonic anhydrase activity prevented short‐term changes in pH.

## Methods

### Ethical approval

The protocol for isolation of porcine airway epithelia was reviewed and approved by the University of Iowa Animal Care and Use Committee. Newborn piglets were obtained from Exemplar Genetics (Exemplar Genetics, Sioux Center, IA) and were euthanized upon arrival by an overdose of Euthasol via cardiac puncture after sedation with 20 mg/kg Ketamine and 2 mg/kg xylazine.

### Isolation, expansion, and culture of porcine airway epithelia

Piglet lungs were excised and proximal large airways, including trachea and main stem bronchi, were dissected out from the airway tree as described (Li et al. 2016). Next, primary porcine airway epithelia were isolated according to an adapted procedure originally developed for tracheal airway cells. Isolated large airway cells were expanded as recently described (Li et al. 2016) using a method developed to conditionally reprogram airway epithelial cells (Liu et al. 2012; Suprynowicz et al. 2012). Briefly, large airway cells were cultured in F media in the presence of 10 *μ*mol/L Y‐27632, a ROCK inhibitor, and low passages of irradiated fibroblast feeder cells NIH‐3T3‐J2 obtained from Dr. H. Green's laboratory at Harvard University (Rheinwald and Green 1975) and maintained at 37°C with 5% CO_2_. After two passages of amplification, expanded epithelial cells were separated from feeder cells and seeded onto collagen‐coated, semipermeable membranes (Corning #3470) at density of 10^6^ cells/cm^2^ and cultured at the air–liquid interface 37°C in a 5% CO_2_ atmosphere as previously described (Zabner et al. 1996) for 2–3 weeks in the absence of feeder cells and ROCK inhibitor. In the first week of seeding at the air–liquid interface, cells were maintained in Small Airway Growth Media (Lonza, Basel, Switzerland) supplemented with 10 ng/mL keratinocyte growth factor for 1 week, after which cells were maintained in USG media. We used the expanded epithelial cells between passage 2 and passage 5 to obtain consistent results.

### Tissue RNA isolation and PCR

Tissue samples from large airway epithelial cells, kidney, intestine, and salivary gland were fixed and stored frozen in RNAlater solution (Invitrogen) followed by thawing and three washes of PBS without divalent cations. Next, the tissues were homogenized in TRIzol reagent solution (Invitrogen) and RNA was extracted using Invitrogen's PureLink RNA Mini Kit according to the manufacturer's protocol. The samples were then processed to cDNA by loading 1000 ng of RNA from each sample using SuperScript IV VILO Master Mix (Invitrogen) according to the manufacturers’ protocol. cDNA concentrations of each sample were quantified using NanoDrop 2000 (ThermoFisher), then diluted to 500 ng/*μ*L. Using Kapa HiFi HotStart ReadyMix PCR Kit a reaction mixture for PCR was made according to the manufacturer protocol using 100 ng of cDNA from each sample with each of the following primer sets for membrane‐bound CA isoforms;

CA‐IX F: 5′‐TCCCACCACAGGAGGAGATT‐3′ IX R: 5′‐ATCCCTGGGAGCCTCAGTAG‐3′

CA‐XII F: 5′‐CCCACTCAACGGATCCAAGT‐3′ XII R: 5′‐CAAACTGCTGGTTGGCAGTC‐3′

Pig Beta Actin F: 5′‐CTGCGGCATCCACGAAAC‐3′ R: 5′‐GTGATCTCCTCCTGCATCCTGTC‐3′

The thermocycler was programmed with the following settings: Initial denaturation of 3 min at 95°C, followed by 25 cycles of denaturation for 20 sec at 98°C, annealing for 15 sec at 63°C, and extension for 15 sec at 72°C; finishing with a final extension of 1 min at 72°C. PCR product was run using electrophoresis on a 5% agarose gel.

### pH_ASL_ measurements

pH_ASL_ was measured using the ratiometric fluorescent pH indicator SNARF‐1 conjugated to dextran (Molecular Probes, Eugene, Oregon), as previously reported (Pezzulo et al. 2012). SNARF has an estimated response time of ~60 msec (Chen et al. 2010b), which is faster than observed pH changes in this study (10 sec to 5 min). SNARF‐1 dextran was applied to the airway–surface liquid as a finely strained powder and 2 h later epithelia were placed in a humidified chamber with 5% CO_2_/21% O_2_/balanced N_2_ heated to 37°C and examined by confocal microscopy (Zeiss 510 Meta NLO). pH was calculated as previously described (Pezzulo et al. 2012), but using a nonlinear fit, as pH_ASL_ was not completely within the linear range of the dye in this study (e.g., when exposed to apical air). Calibration curves were fitted in GraphPad Prism with a modified acid‐base titration equation (Boyarsky et al. 1988):

$$\text{ratio observed}=a+b\;\;\left(\frac{10^{\left(\text{pH}-\text{pKa}\right)}}{1+10^{\left(\text{pH}-\text{pKa}\right)}}\right)$$

Where variables *a* and *b*, as well as pKa, are obtained from the fit. The *ratio observed* is the recorded 580/640 emission. pH and pKa have their classic definitions. In this study, SNARF‐1 dextran had a pKa of 7.44 consistent with 37°C values reported by various groups (Buckler and Vaughan‐Jones 1990; Blank et al. 1992; Westerblad et al. 1997; Ch'en et al. 2003). In some experiments, the CO_2_ was changed from 5% CO_2_ to air (compressed air containing 0.04% CO_2_) every 4 min and the change in pH_ASL_ was monitored in real time as described above. To test carbonic anhydrase activity, cells were pretreated with 20 *μ*mol/L acetazolamide (Sigma‐Aldrich, St. Louis, MO), a carbonic anhydrase inhibitor, or 1.5 U carbonic anhydrase isozyme II (Sigma‐Aldrich, St. Louis, MO), in a final volume of 0.5 *μ*L on the apical surface, then pH_ASL_ was monitored as described above.

### Kinetic pH_ASL_ analysis

The experimental data (*n* = 5–8 independent experiments performed on cells isolated from *N* = 3–4 pigs) were fitted using the least‐squares method in GraphPad Prism with the following equations:

Association phase (removal of CO_2_):

$$\begin{array}{c} {\text{pH}}_{\mathrm{ASL}}=\;\left\{\begin{array}{c} t\;\leq t_{0};\;pH_{{\mathrm{ASL}}_{0}}\;\\ t>t_{0};\;\;pH_{{\mathrm{ASL}}_{0}}+\left(\;pH_{{\mathrm{ASL}}_{\max}}-\;pH_{{\mathrm{ASL}}_{0}}\right)\\ \times \left(1-e^{-k\;\times \;\left(t-t_{0}\right)}\right) \end{array}\right. \end{array}$$

Decay phase (addition of CO_2_):

$$\begin{array}{c} pH_{\mathrm{ASL}} \end{array}=\;\left\{\begin{array}{c} t\;\leq t_{0};\;pH_{{\mathrm{ASL}}_{0}}\\ t>t_{0};\;\;pH_{{\mathrm{ASL}}_{\min}}+\left(\;pH_{{\mathrm{ASL}}_{0}}-\;pH_{{\mathrm{ASL}}_{\min}}\right)\\ \times e^{-k\;\times \;\left(t-t_{0}\right)} \end{array}\right.$$

Where pH_ASL_ is the instantaneous ASL pH, *t* is the instantaneous time, *t*_0_ is the time of the removal (association equation) or addition (decay equation) of CO_2_, $\;{\text{pH}}_{{\mathrm{ASL}}_{0}}$ is the baseline *pH*_*ASL*_, $\;{\text{pH}}_{{\mathrm{ASL}}_{\max}}$ is the fitted maximal *pH*_*ASL*_ (association equation only), $\;pH_{{\mathrm{ASL}}_{\min}}$ is the fitted minimum pH_ASL_ (decay equation only), and *k* is the obtained rate constant for the fit. *R* ^2^ values were >0.98 for all fitted data.

To calculate the mean pH, the following equation was used:

$${\text{pH}}_{\mathrm{mean}}=\;-\log\left(\frac{10^{-pH_{1}}+\;10^{-pH_{2}}}{2}\right)$$

Where pH_mean_ is the mean pH of the proton activities of pH_1_ (steady state under 5% CO_2_) and pH_2_ (steady state under air).

To determine the rate constants for large airway epithelia exposed to acetazolamide, carbonic anhydrase, or control treatment, the data were fitted using the least‐squares method. Consistent with carbonic anhydrase being an enzyme, we observed that equilibrium pH_ASL_ values for each experiment were not different. Therefore, we could compare rate constants among these three treatments.

### Simulation of pH_ASL_ changes in the respiratory cycle

A program to calculate real‐time pH_ASL_ changes during the respiratory cycle was coded in MATLAB version R2015a (Mathworks) and is freely available upon request. User‐defined input values are the respiration rate, inspiratory:expiratory (I:E) ratio, initial pH_ASL_, and fitted curves obtained using the association and decay equations described above. The program uses pH‐dependent rates, which accommodates for pH‐dependent unknown variables (e.g., intrinsic buffering capacity) to predict pH_ASL_ values in the time domain. Briefly, the program computes the rate of pH change (*d*pH/*d*t) along the fitted inspiration curve for a user‐defined initial pH value and outputs a computed pH with a user‐defined time binning (10 points for the smallest respiratory phase in this study). This process is looped for each bin until the end of inspiration is achieved. After completing the inspiration phase, the program begins the same process on the expiratory curve initiating at the final pH of the inspiration phase. The program then loops between these two phases until a user‐defined simulation time is reached. Data were output to a Microsoft Excel file. From these simulations, the mean oscillatory pH (pH_OSC_) and the root mean square (RMS) of the oscillatory pH (pH_RMS_) were computed. The pH_RMS_ was calculated using the RMS equation for a triangular wave:

$$pH_{\mathrm{RMS}}\;=\;a\;/\sqrt{3}$$

Where *a* is the amplitude of the triangular wave. In some simulations, the steady‐state simulation was acidified or alkalinized and the time to recovery to baseline was monitored and fitted with the association (acid addition) or decay equations (alkaline addition) defined above in prism to obtain time constants (τ).

### Statistics

Data are expressed as mean ± SEM or regressions fitted by the least‐squares method. A *P* value less than 0.05 was accepted as significant. Means were compared using ANOVA with Bonferroni or Dunnet correction (for comparing acetazolamide and carbonic anhydrase application to control), and unpaired, or paired t‐tests where appropriate. Statistical significance among regressions was computed by comparing the residual sum‐of‐squares to obtain an F ratio. P values were obtained in Excel for a given F ratio with corresponding degrees of freedom.

## Results

### Rate of pH_ASL_ change in response to CO_2_

**Figure 1:**
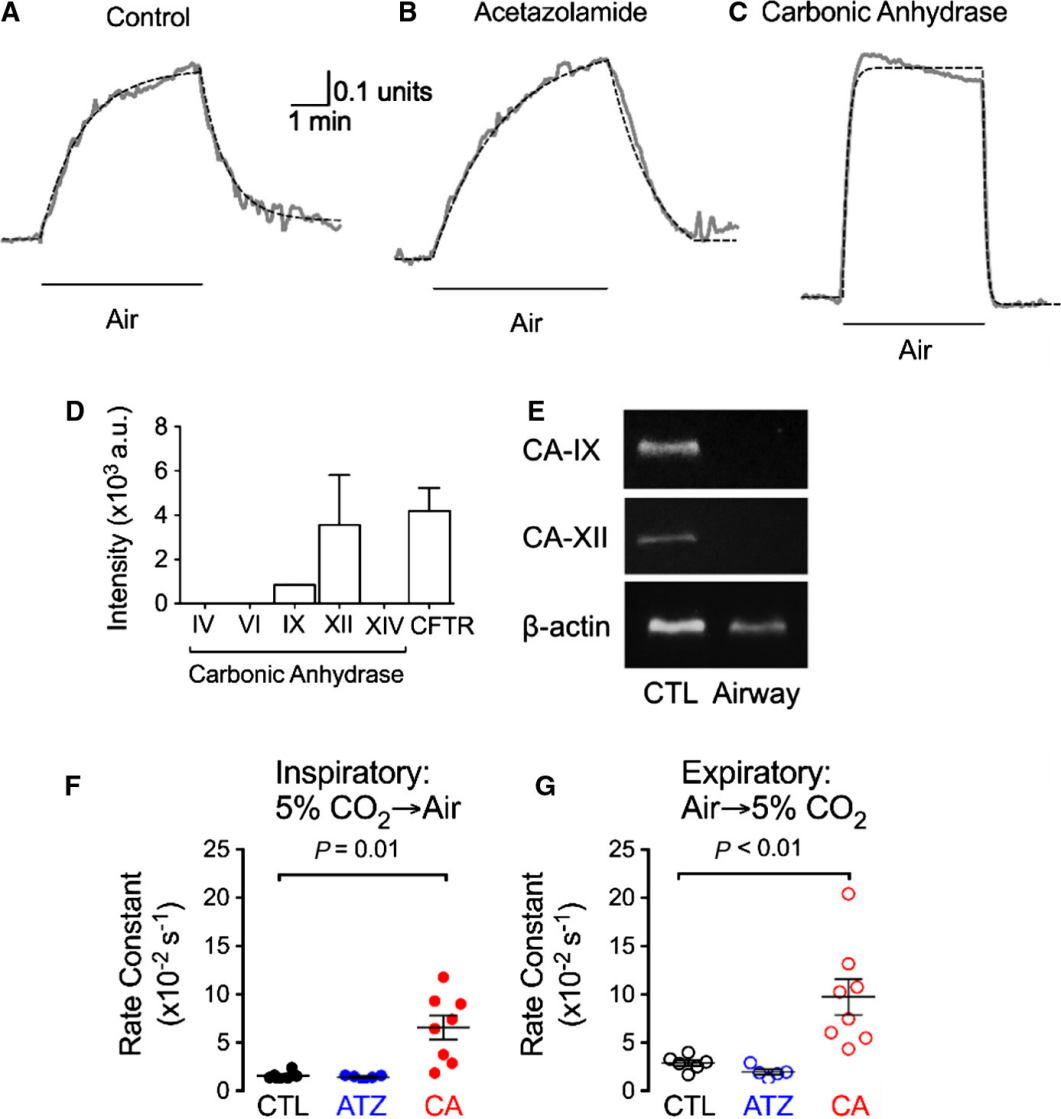
Alterations in pH_ASL_ for large airway cultures in response to changes in CO_2_. A–C, Cells were exposed bilaterally to 5% CO_2_, followed by an apical shift to air (noted on traces as “Air”). After monitoring pH_ASL_ for 5 min, apical gas was subsequently shifted back to 5% CO_2_. These maneuvers changed pH_ASL_ from ~7.2 to ~7.7 in each condition. Each experiment was fitted (black‐dotted lines) using the exponential equations described in the Methods. (A) Control conditions. (B) Cells were pretreated with 20 *μ*mol/L acetazolamide, a carbonic anhydrase inhibitor, on the apical surface. (C) Cells were pretreated with 1.5 U carbonic anhydrase on the apical surface. Experiments were performed on matched samples isolated from the same animal. Each trace is a representative example of one experiment. (D) RNA sequencing microarray data for large airway epithelial cells cultured at the air–liquid interface. (E) Pig trachea RT‐PCR for CA IX (control: intestine) and CA XII (control: renal cortex). (F and G) Average rate constants for airway epithelia when CO_2_ was shifted from (F) 5% CO_2_ to air or (G) air to 5% CO_2_. Each dot represents one culture. CTL: control; ATZ: acetazolamide; CA: exogenous carbonic anhydrase.

To assess how changes in CO_2_ affect pH_ASL_, we studied differentiated cultures of porcine airway epithelium. We varied apical CO_2_ between 5% and 0.04% (air) while maintaining basolateral CO_2_ at 5%. Switching from 5% CO_2_ to air led to a slow pH_ASL_ change that plateaued after ~4 min (Fig. 1A). This change was reversible upon reexposure to 5% CO_2_. These changes occurred with a time constant of approximately 50 sec.

The reaction CO_2_ + H_2_O ↔ H^+^ + HCO_3_^–^ is catalyzed by carbonic anhydrase (Kim et al. 2014). Because the observed pH change is slow, we predicted that carbonic anhydrase activity is low within intact ASL. We first assessed mRNA transcripts of carbonic anhydrase isozymes (*Ca*) predicted to be enzymatically active outside of cells, which include the membrane‐bound and secreted carbonic anhydrase isozymes. Microarray analysis from Li et al. 2016 (Fig. 1D) revealed that most mRNA transcripts for these isozymes were present at levels lower than *Cftr* mRNA transcripts, which is present in low abundance in airway epithelia (Trapnell et al. 1991; Chu et al. 1992). *CaXII* mRNA transcripts were present at similar levels to *Cftr*, however, carbonic anhydrase XII protein has low enzymatic activity (Tureci et al. 1998). We also investigated the expression of *CaIX* and *CaXII* in pig trachea by RT‐PCR. As shown in Figure 1E *CaIX* and *CaXII* are expressed in intestine and renal cortex tissues, respectively, but not in pig trachea.

To test the hypothesis that ASL upon differentiated airway cultures has minimal carbonic anhydrase activity, we performed two experiments. We added the carbonic anhydrase inhibitor acetazolamide and found it did not affect pH_ASL_ kinetics induced by apical CO_2_ changes (Fig. 1B). In contrast, adding recombinant carbonic anhydrase to the ASL accelerated the rate of pH_ASL_ change in response to changes in apical CO_2_ (Fig. 1C). Rate constant analysis (Fig. 1F and G) suggests that airway epithelia lack substantial carbonic anhydrase activity resulting in slow changes in pH_ASL_ when CO_2_ concentration changes. These slow changes would minimize the change in pH_ASL_ during breathing.

### Computational modeling of dynamic pH_ASL_ changes in airway epithelia

To further explore how respiratory cycle properties (e.g., respiratory rate and inspiratory:expiratory (I:E) ratio) affect pH_ASL_, we performed computational modeling using the pH‐dependent rates obtained from Figure 1. This approach models the real‐time pH changes and allows for integration of the pH‐dependent processes (e.g., ASL buffering capacity).

**Figure 2:**
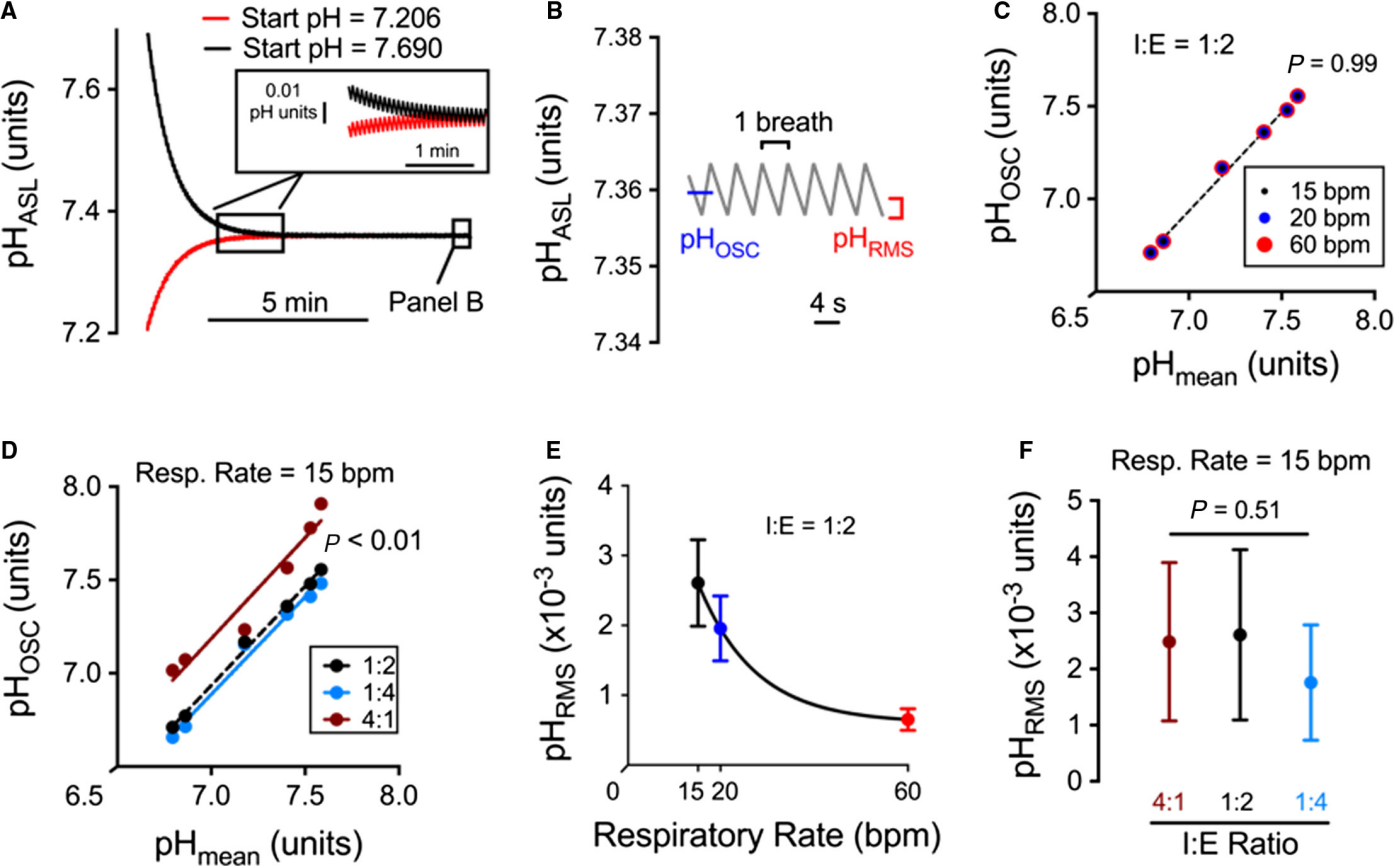
Airway pH_ASL_ is more alkaline during the respiratory cycle than in the constant presence of 5% CO_2_, with minimal breath‐to‐breath fluctuations. (A) Modeling pH_ASL_ changes during respiratory cycles with a 15 breath per minute (bpm) respiratory rate and 1:2 I:E ratio. Beginning the simulation at varying pH values does not change steady‐state pH achieved. (B) Expansion of the steady‐state pH changes from panel A. Parameters that correspond to the mean pH during steady‐state oscillation (pH_OSC_) and the bandwidth of the pH oscillation (pH_RMS_) are noted. (C) The I:E ratio was held constant at 1:2, and the respiratory rate was modeled at 15, 20, and 60 bpm. Because pH_OSC_ will be influenced by the steady‐state pH values of a given culture, simulated pH_OSC_ was plotted as a function of the midpoint H^+^ activity between 5% CO_2_ and air. There was no change in pH_OSC_. (D) The respiratory rate was held constant 15 bpm (typical human breathing cycle), and the I:E ratios were modeled at 1:2 (physiologic ratio), 1:4 (obstructive airway disease), and 4:1 (airway pressure release ventilation). The pH_OSC_ is directly correlated with relative phase lengths. (E and F) pH_RMS_ varied inversely with the respiration rate (E) and was not affected by the I:E ratio (F). bpm: breaths per minute. (C and D) Filled circles represent a simulation for rates obtained from different cultures. (E and F) Error bars represent standard error of simulations performed using rates obtained from different cultures.

The initial model parameters included a 15 breath per minute (bpm) respiratory rate and an I:E ratio of 1:2, which reflects a typical human respiratory cycle (Cheng et al. 2010). To validate the model, we performed simulations using data beginning at the minimum pH_ASL_ value (i.e., the pH_ASL_ for 5% CO_2_) or the maximum pH_ASL_ value (i.e., the pH_ASL_ for air) for each culture. Example simulations for one culture are shown in Figure 2A. For these simulations, we quantified the magnitude of the pH oscillations as root‐mean‐squared values (pH_RMS_) and the mean pH during steady‐state oscillations (pH_OSC_) (Fig. 2B). For the experiment shown, a pH_OSC_ of 7.36 was reached after ~4 min with little oscillation (pH_RMS_ = 0.002 pH units). These simulation data suggest that the pH_ASL_ is more alkaline during the respiratory cycle than in the constant presence of 5% CO_2_, under which most airway culture studies are performed, and the pH does not fluctuate appreciably from breath to breath.

Next, we modeled changes in the respiratory rate (15, 20, and 60 breaths per minute) and the I:E ratios to mimic physiologic or pathologic scenarios (1:2 – physiologic ratio, 1:4 – obstructive airway disease, and 4:1 – airway pressure release ventilation). pH_OSC_ during steady state was unaffected by the respiratory rate (Fig. 2C) and was directly correlated to the I:E ratio (Fig. 2D). pH_RMS_ was inversely correlated to respiratory rate (Fig. 2E), but unaffected by the I:E ratio (Fig. 2F). In summary, the I:E ratio affects the mean steady‐state pH (pH_OSC_) and the respiration rate affects the magnitude of pH oscillations (pH_RMS_).

**Figure 3:**
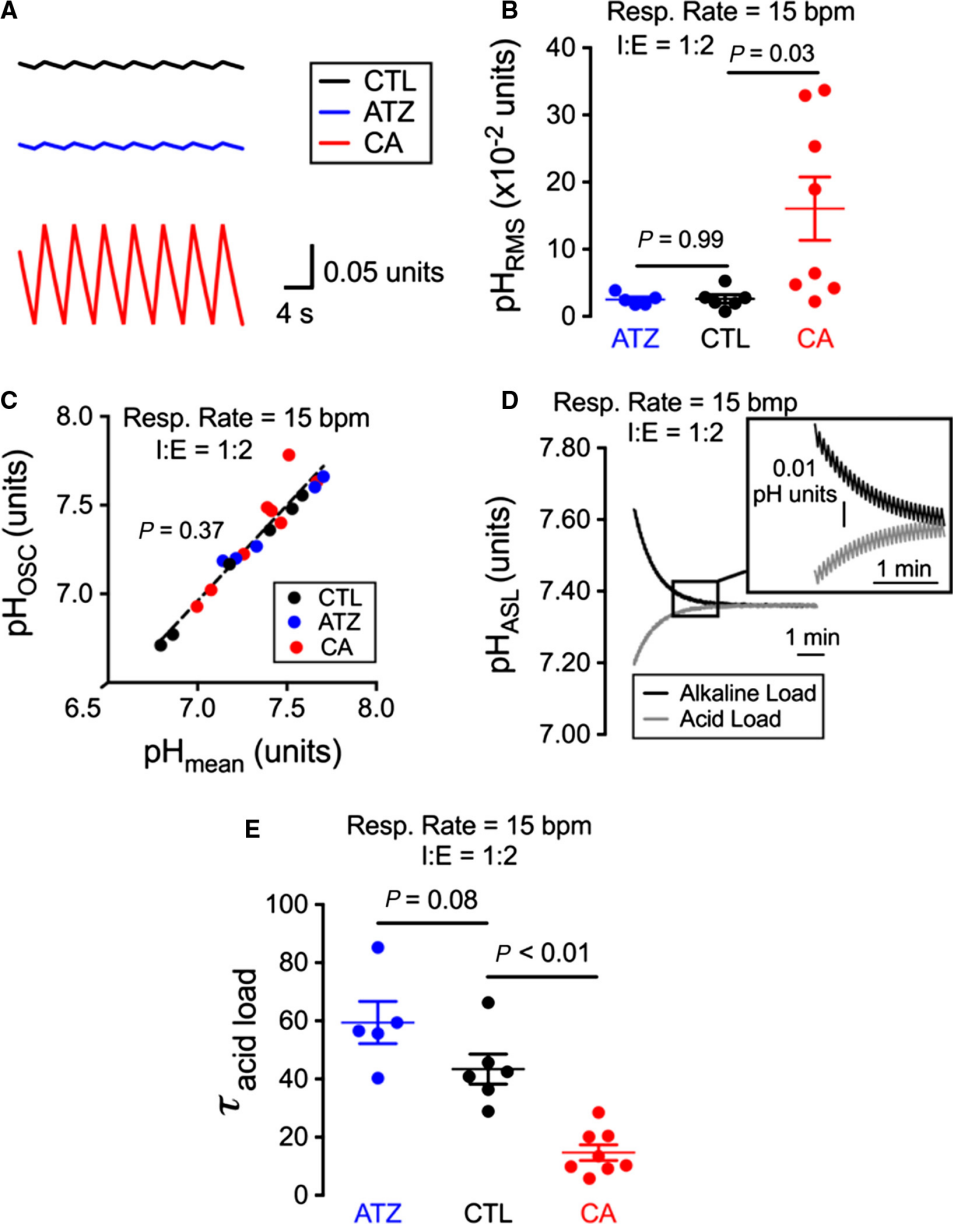
Modeling changes in pH_ASL_ demonstrates that carbonic anhydrase activity appreciably increases predicted pH oscillations during breathing. (A) Modeling pH_ASL_ changes under control conditions (CTL) or in response to acetazolamide (ATZ) or carbonic anhydrase treatment (CA). (B and C) Modeling the effect of acetazolamide or carbonic anhydrase on (B) pH_OSC_ or (C) pH_RMS_. Carbonic anhydrase promoted a significant increase in pH_RMS_, whereas carbonic anhydrase had no effect on pH_OSC_. (D) Modeling pH_ASL_ recovery from a 20 nmol/L acid or alkaline load. (E) Time to recovery (*τ*) from a 20 nmol/L acid load under control conditions or in the presence of acetazolamide or CA with a respiratory rate of 15 breaths per minute and an I:E ratio of 1:2. CA but not acetazolamide significantly accelerated the time to recovery. CTL, control; ATZ, acetazolamide; CA, exogenous carbonic anhydrase; bpm, breaths per minute. (B, D, E) Filled circles represent a simulation for rates obtained from different cultures.

Based on our rate analysis, exogenous carbonic anhydrase would increase pH oscillations. To demonstrate this, the simulation was repeated using rates obtained when we added exogenous carbonic anhydrase, vehicle, or acetazolamide (Fig. 3A). The presence of carbonic anhydrase increased ASL pH_RMS_ (Fig. 3B), but not steady‐state pH_OSC_ (Fig. 3C) relative to controls. These results were expected because adding carbonic anhydrase enzymatic activity should alter the pH kinetics, but should not alter the pH equilibrium achieved.

Finally, we modeled pH_ASL_ recovery from simulated acid or alkaline addition to the ASL (Fig. 3D). We found no difference in recovery to an effective 20 nmol/L acid or alkaline addition under control conditions or in the presence of acetazolamide, whereas applying carbonic anhydrase significantly accelerated the time to recovery (Fig. 3E). These data indicate that lack of appreciable airway carbonic anhydrase activity in the ASL would minimize pH_ASL_ changes during respiration and exogenous carbonic anhydrase may help acidic airways achieve a more alkaline pH, at least for a fraction of the respiratory cycle (i.e., flanking peak inspiration).

## Discussion

Because the large airway is exposed to oscillations in CO_2_ during the respiratory cycle, our goal was to understand how varying CO_2_ concentration affects the pH_ASL_ on differentiated airway epithelia maintained at the air–liquid interface. We found that, after altering CO_2_, the time to plateau was on the order of minutes. In contrast, respiratory cycles occur within seconds and therefore pH_ASL_ changes are small during breathing. The slow change in pH_ASL_ was a result of low carbonic anhydrase activity of the ASL. These data are consistent with previous reports that ASL removed from epithelia has minimal carbonic anhydrase activity (Candiano et al. 2007; Kim et al. 2014). Thus, loss of carbonic anhydrase activity in the bulk ASL may represent an evolutionary selection to minimize dynamic changes in pH_ASL_ in response to respiration.

At least 16 different carbonic anhydrase isoforms have been described in mammals (Supuran et al. 2008; Imtaiyaz Hassan et al. 2013). The most relevant isoforms to this study are the membrane‐bound and secreted forms, including carbonic anhydrase IV, VI, IX, XII, and XIV (Pinard et al. 2015). Carbonic anhydrase IX has been observed in human airways, but only under hypoxic conditions (Polosukhin et al. 2007, 2011). Carbonic anhydrase VI, also known as gustin, is the only isoform that is secreted, and it is expressed highly in the salivary glands (Thatcher et al. 1998). Carbonic anhydrase VI has been found by immunocytochemistry in rat lung (Leinonen et al. 2004) and by mRNA detection in human neuroendocrine bodies (Livermore et al. 2015), but not on surface epithelia of the airways. Carbonic anhydrase XIV was the only membrane‐bound isozyme detected at the mRNA level in human infant lung epithelium (Livermore et al. 2015). Recent immunohistochemistry data suggest that carbonic anhydrase XII is expressed in the terminal bud of human respiratory epithelial cells (Lee et al. 2016). Mutations in carbonic anhydrase XII have been implicated in lung disease (Lee et al. 2016), but the mechanism remains unclear. Regardless, we speculate that carbonic anhydrase XII is not likely to regulate pH_ASL_ because carbonic anhydrase XII's subcellular localization suggests lack of direct contact with the ASL (Lee et al. 2016). In addition, carbonic anhydrase XII has low enzymatic activity that is 15% of carbonic anhydrase IV (Tureci et al. 1998). This notion is consistent with our data that the ASL of large airways has minimal carbonic anhydrase activity.

Respiratory rates vary in health and disease. The average respiratory rate in healthy resting humans is approximately 15–20 bpm. During exercise or during respiratory failure, the respiratory rate can increase to 60 bpm. Our modeling data suggest that these changes would have minimal effect on pH_ASL_. In patients with obstructive lung disease, such as asthma or chronic obstructive pulmonary disease (COPD), the expiratory phase is increased. Our data suggest that the decrease in pH_ASL_ due to the I:E ratio would be modest (~0.1 pH units). Inverse ratio ventilation is routinely used in intensive care units to ventilate patients that do not respond to conventional mechanical ventilation (Cole et al. 1984). Our model showed that an I:E ratio of 4:1 would significantly increase pH_ASL_.

Why would the airway evolve to lack carbonic anhydrase in the ASL? Based on the Henderson‐Hasselbalch equation, fluctuations in CO_2_ from 5% to 0.04% in the presence of 20 mM HCO_3_^–^ and carbonic anhydrase could result in a pH change from 7.4 to 9.5. Thus, we speculate that the large airway evolved to have minimal carbonic anhydrase activity and nonbicarbonate buffering capacity to mitigate extreme alkaline shifts in pH_ASL_.

Additionally, airway lacking carbonic anhydrase may have been selected for based upon beneficial pH‐dependent enzymes, such as *β*‐defensin‐3 and LL‐37 (Abou Alaiwa et al. 2014b). Simulations with carbonic anhydrase (Fig. 3A) have a pH oscillation magnitude of ~0.1 unit, which could affect enzyme function. For example, consider an enzyme whose function is defined by a titration curve and pKa equals that of the midpoint of the ASL pH oscillation. Then an oscillation of 0.1 (midpoint ± 0.05 units) would undergo a ~11.5% change in protonation from peak to peak. In contrast, a pH change of 0.01 (midpoint ± 0.005 units), as seen in the absence of exogenous carbonic anhydrase (Fig. 3A), would change the theoretical enzyme protonation state by only ~1% from peak to peak.

Our study also has limitations. First, we did not remove mucus from cultured epithelia. However, mucus is present in vivo and it is proposed to have only a minor role in buffering at the physiological pH_ASL_ values (Holma and Hegg 1989; Kim et al. 2014). During inhalation, where CO_2_ is low, pH is ~7.8, and therefore HCO_3_^–^ is ~500 *μ*mol/L, high concentrations of mucus may provide additional buffering capacity through its free cysteines (pKa ~8.5). Second, the model does not take into consideration submucosal glands that are present in an intact lung. However, acidic submucosal gland secretions (Widdicombe and Wine 2015) are anticipated to be similar to the perturbations associated with acid loading modeled in Figure 3. Third, prolonged changes in apical CO_2_, from which our rates were obtained, may have a modest effect on the intracellular pH under the apical membrane. Fourth, additional buffering capacity may be excreted in vivo by the epithelium or the presence of other cell types (e.g., leukocytes during disease). None of these limitations would affect our conclusion that pH_ASL_ changes were slow in response to CO_2_ due to nominal carbonic anhydrase activity.

In summary, pH_ASL_ oscillations in the respiratory cycle are small, avoiding extreme pH values that may be caustic to airway epithelia. However, given that pH_ASL_ is more acidic in many airway diseases (e.g., in CF due to decreased HCO_3_^–^ secretions) and contributes to host defense defects, delivering or increasing ASL carbonic anhydrase may be useful to increase pH_ASL_. An increase in carbonic anhydrase activity would increase the pH oscillation magnitude, achieving more alkaline pH values for a fraction of the respiratory cycle.

## Conflict of Interest

The University of Iowa has licensed CF pigs to Exemplar Genetics, and MJW receives royalties from the license. No conflicts of interest, financial, or otherwise are declared by the other authors.
